# Colorimetric Detection of *Escherichia coli* O157:H7 with Signal Enhancement Using Size-Based Filtration on a Finger-Powered Microfluidic Device

**DOI:** 10.3390/s20082267

**Published:** 2020-04-16

**Authors:** Younggeun Jo, Juhwan Park, Je-Kyun Park

**Affiliations:** 1 Department of Bio and Brain Engineering, Korea Advanced Institute of Science and Technology (KAIST), 291 Daehak-ro, Yuseong-gu, Daejeon 34141, Korea; ygjo@kaist.ac.kr (Y.J.); juhwan3275@kaist.ac.kr (J.P.); 2 KAIST Institute for Health Science and Technology, 291 Daehak-ro, Yuseong-gu, Daejeon 34141, Korea

**Keywords:** colorimetric detection, *E. coli* O157:H7, finger-powered, microfluidics, size-based filtration

## Abstract

Although immunomagnetic separation is a useful sample pretreatment method that can be used to separate target pathogens from a raw sample, it is challenging to remove unbound free magnetic nanoparticles (MNPs) for colorimetric detection of target pathogens. Here, size-based filtration was exploited for the rapid on-site detection of pathogens separated by immunomagnetic separation in order to remove unbound free MNPs using a finger-powered microfluidic device. A membrane filter and an absorbent pad were integrated into the device and a mixture of unbound free MNPs and MNP-bound *Escherichia coli* (*E. coli*) O157:H7 was dispensed over the membrane filter by pressing and releasing the pressure chamber. A colorimetric signal was generated by MNP-bound *E. coli* O157:H7 while unbound free MNPs were washed out by the absorbent. Furthermore, the colorimetric signals can be amplified using a gold enhancer solution when gold-coated MNPs were used instead of MNPs. As a result, 10^2^ CFU/mL *E. coli* O157:H7 could be detected by the enhanced colorimetric signal on a proposed device.

## 1. Introduction

Foodborne diseases are a national issue not only in undeveloped countries but also in advanced countries. In the United States, foodborne pathogens caused 9.4 million foodborne diseases, 55,961 hospitalizations, and 1351 deaths annually [1]. Because foodborne pathogens proliferate rapidly, early-stage screening of the pathogens is important to prevent serious diseases. As interest in well-being foods has increased recently, there is an increasing demand for uncooked foods at high temperatures. It is therefore important to rapidly screen the foodborne pathogens on-site. The traditional method based on culture-based direct plating is still the “gold standard” for the detection and identification of pathogens [2,3]. This direct plating method provides information on the number of pathogens in food samples at relatively low prices with good specificity and sensitivity. However, because this method visually selects presumptive positive colonies on an agar plate, morphologically similar competitive microflora are often selected and grown with target pathogens in agar media. Therefore, this method requires highly skilled technicians and various cultivation processes for enrichment, isolation, and identification, which takes 3–5 days for the detection of the pathogens.

To overcome these limitations, several methods have been reported for rapid detection without the cultivation process. For rapid detection, these methods utilized highly sensitive detection methods such as surface plasmon resonance [4,5], surface-enhanced Raman scattering (SERS) [6,7], polymerase chain reaction (PCR) [8,9], electrochemical impedance spectroscopy [10], and fluorescence spectroscopy [11]. Furthermore, there have been a number of label-free bacteria detection methods using a nanoparticle-induced colorimetric signal or SERS signal [12,13,14,15]. As the labeling process is unnecessary, overall sample preparation procedures can be simplified, but it is difficult to specifically detect target bacteria due to the limitations of the label-free methods. Although the above methods can detect bacteria with high sensitivity, there are needs on signal analysis tools as well as a pretreatment process requiring bulky and benchtop equipment that can make the overall detection system complicated.

On the other hand, for the rapid and simple pretreatment process for purification of the pathogens on-site, immunomagnetic separation has been widely used. Immunomagnetic separation uses antibody-conjugated magnetic nanoparticles (MNPs) to separate the target bacteria from the food matrix by immunoreaction with the bacteria under the magnetic field. Immunomagnetic separation can simply separate bacteria from the food matrix, but the use of immunomagnetic separation often causes analytical errors when unbound free MNPs coexist in the bacterial separation process.

To overcome this limitation, several methods have been reported by conjugating additional nanoparticle labels (e.g., gold nanoparticle for the SERS signal [6,7,16], a bigger magnetic particle for the magnetic relaxation time signal [17], quantum dot for the fluorescent signal [18]) with the target bacteria. In addition, pathogens were detected by separating unbound free MNPs using a hydrophoresis-based microfluidic device [19] or by size-based filtration [20]. However, these methods require laborious equipment to read out the result signal or to separate unbound free MNPs, so that they are insufficient for the on-site detection method.

Meanwhile, the finger-powered microfluidic devices can simply control the flow on-demand by pressing and releasing the buttons with fingers, so that they have been applied in various fields, including pre-transfusion tests [21,22], droplet generation [23], colorimetric glucose assay [24], and CD4+ cell counting for human immunodeficiency virus diagnostics [25]. In this study, a finger-powered microfluidic device was applied for the rapid and on-site detection of *Escherichia coli* (*E. coli*) O157:H7. To separate unbound free MNPs and to detect MNP-bound *E. coli* O157:H7 by colorimetric signals, a membrane filter is integrated into the finger-powered microfluidic device. By actuating the buttons, reagents are dispensed into the filtration region and unbound free MNPs are removed by the absorbent under the membrane filter. When gold-coated MNPs are used, the colorimetric signals from the filtered MNPs bound to *E. coli* O157:H7 are amplified by injecting a gold enhancer solution.

## 2. Materials and Methods

### 2.1. Device Fabrication

**Figure 1:**
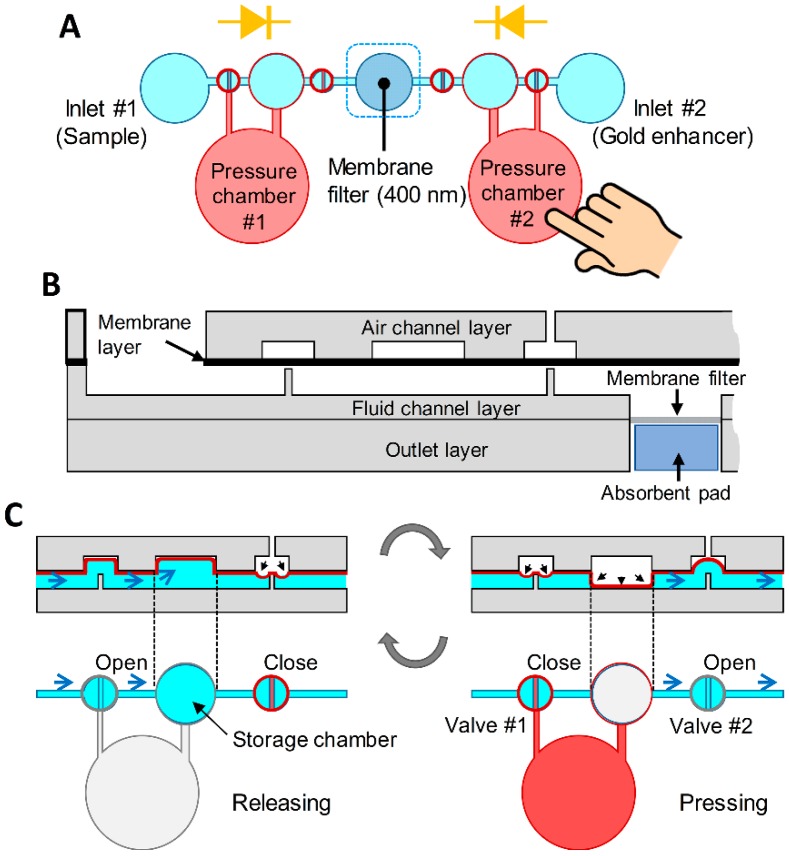
Design and working principle of the device. (**A**) Schematic of the device. (**B**) A membrane filter and an absorbent pad were embedded in the device to wash out unbound free magnetic nanoparticles (MNPs). (**C**) Working principle of the finger-powered microfluidic pump when the pressure chamber is pressed or released.

A finger-powered microfluidic device (70 mm (*w*) × 30 mm (*l*) × 9 mm (*h*)) was fabricated by soft lithography as previously described [21,22] and a polycarbonate track-etched membrane filter (GE Healthcare, Buckinghamshire, UK) was integrated into the device by using an uncured poly(dimethylsiloxane) (PDMS) as an adhesive. In more detail, the device consisted of four PDMS layers; an air channel layer, membrane layer, fluid channel layer, and outlet layer (Figure 1B). First, an air channel layer (height of the air channel = 40 μm) was bonded with a membrane layer (thickness = 25 μm). The membrane layer was obtained by spin-coating the uncured PDMS on a bare silicon wafer at a speed of 1500 rpm for 60 s and incubation on a hot plate at 150 °C for 10 min. After bonding the two layers, the assembled layers were incubated in an oven at 65 °C for a few hours. During the incubation, another layer was prepared. When a fluid layer (height of the fluid channel = 100 m) was bonded with an outlet layer, a membrane filter was inserted between two layers. For robust bonding, the layers and the membrane filter were sequentially stacked after stamping on thinly spread uncured PDMS prepared by spin coating. The stacked layers were incubated in an oven at 65 °C for a few hours. And then, the upper layers (air channel layer and membrane layer) were boned with the bottom layers (fluid layer and outlet layer). Finally, an absorbent pad (Grade 222, Alshorm, Finland) was inserted into the outlet.

### 2.2. Sample Preparation

Carboxyl functionalized MNPs (Creative Diagnostics, Shirley, NY, USA) were conjugated with anti-*E. coli* O157:H7 antibody (Sigma-Aldrich, St. Louis, MO, USA) using the covalent coupling of the antibody by activating the carboxyl groups with water-soluble carbodiimide. The carbodiimide reacts with the carboxyl group to create an active ester that is reactive to primary amines on the antibody. The conjugation procedure used in this paper is as follows. Carboxyl functionalized MNPs were washed twice with distilled water. After the second wash, the MNPs were dispersed using ultrasound to prevent clustering. The MNPs were then re-suspended in 450 μL of 0.1 M 2-(*N*-morpholino) ethanesulfonic acid (MES) buffer (pH 5.2) and sonicated for 1 min. Then, 150 μL of 460 mM sulfo-N-hydroxysulfosuccinimide (sulfo-NHS) (Sigma-Aldrich) and 150 μL of 156 mM ethylcarbodiimide hydrochloride (EDC) (Sigma-Aldrich) were added into the MNP solution and incubated with continuous shaking for 30 min at room temperature. After the incubation, the activated nanoparticles were washed by centrifugation with 0.01 M phosphate-buffered saline (PBS) (pH 7.0). PBS buffer containing 450 μg of anti-*E. coli* O157:H7 antibody was added to the activated nanoparticles and incubated for 3 h with continuous shaking at room temperature. After incubation, the MNPs were washed by centrifugation with PBS buffer to eliminate unbounded free antibodies. Then, antibody-conjugated MNPs were re-suspended in a storage solution containing 10 mM Tris (hydroxymethyl) aminomethane (Tris), 0.05% bovine serum albumin (pH 8.0), 0.2% polyethylene glycol (Sigma-Aldrich), 0.2% Tween-20 (Sigma-Aldrich), and 0.2% Triton X-100 (Sigma-Aldrich) to stop the conjugation reaction between antibody and activated MNPs and to minimize clustering of MNPs. And, the MNPs were dispersed through mixing by pipetting up and down before conjugation with *E. coli* O157:H7 and before loading into the device. A gold enhancer solution was purchased from Nanoprobes (Yaphank, NY, USA).

### 2.3. Signal Measurement

After the detection process, the color of the membrane filter changes from white to dark because of the remaining MNPs that do not pass through the membrane filter. For the numerical analysis of the results, the color of the membrane filter was photographed, and the color intensity of the membrane filter of each result image was measured. To eliminate background noise, the photos were captured with the device on white paper using a digital single-lens reflex (DSLR) camera (D7200; Nikon, Tokyo, Japan). The acquired images were analyzed with the ImageJ program (National Institutes of Health, Bethesda, MD, USA). First, the images were converted into a grayscale image and inverted black and white to get positive result signals. Through this process, the color of the membrane filter is changed from black to white in proportion to the MNPs remained on the filter. Consequently, the whiteness of the membrane filter was proportional to the amount of the remained MNPs. And then, the average whiteness of the round region of the membrane filter was measured.

## 3. Results & Discussion

### 3.1. Design and Concept

The device consists of two inlets, one outlet, and two pressure chambers (Figure 1A). The two inlets are used for injection of the sample solution and the gold enhancer solution, respectively, while two pressure chambers are the regions for pressing and releasing with the fingers. The device was designed to induce the fluid flow using a pressure change inside the microchannel caused by repetitive pressing and releasing of the fingers. In addition, the device was designed to prevent the backflow of fluid by allowing the fluid to flow in one direction only. This finger-powered microfluidic device consists of four layers (Figure 1B). The top layer is an air channel designed to transfer the pressure change from the pressure chamber into the fluidic channel by the deflection of the second layer. The second layer is a PDMS membrane layer that moves up and down between the upper air channel and the lower fluid channel by the pressure change, thus acting as a valve and a pump. The third layer was designed as a fluid channel for the transfer of the injected solution from the inlets. The bottom layer includes an outlet with an incorporated absorbent pad. A membrane filter was inserted between the bottom layer and the third layer so that the sample flowing in the device can be separated through the membrane filter into bacteria–MNP complex and unbound free MNPs.

**Figure 2:**
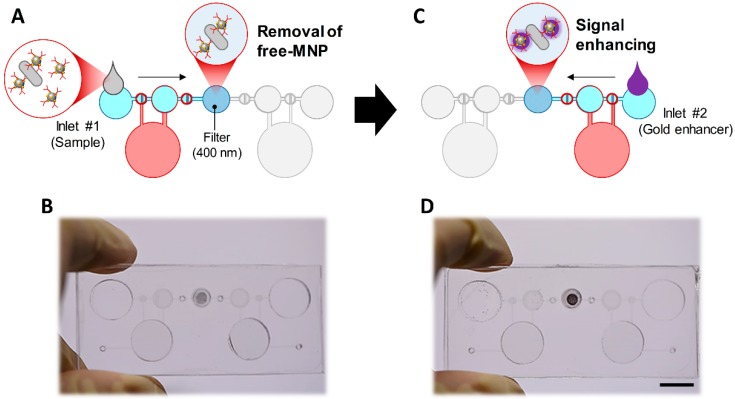
Procedures for the device operation. (**A**,**B**) Unbound free MNPs were washed out and MNP-bound *E. coli* O157:H7 generates a colorimetric signal on a filter membrane. (**C**,**D**) The colorimetric signal was amplified by injecting a gold enhancer solution. Scale bar = 1 cm.

Two valves and one storage chamber were designed to transfer the fluid from one inlet to the membrane filter inside the device (Figure 1C). First, when the pressure chamber is pressed by the finger, the PDMS membrane in the storage chamber is compressed by positive pressure, and the air existing in an initial state of the storage chamber is discharged as valve #2 is opened and valve #1 is closed. And then, when the pressure chamber is released, the PDMS membrane is lifted by the negative pressure of the pressure chamber, and the resulting negative pressure in the storage chamber causes the solution from the inlet to enter the storage chamber through the opened valve #1. At this stage, as valve #2 is closed due to the negative pressure in the storage chamber, the solution only comes from the inlet without the backflow from the outlet. When the pressure chamber is again pressed by the finger, the PDMS membrane in the storage chamber is compressed by positive pressure, and the solution in the storage chamber is discharged through opened valve #2 as valve #1 is closed. By repeating this process, the solution moves from the inlets to the membrane filter. To prevent the sample solution from flowing into the direction of inlet #2, the pressure chamber #2 is always pressed with the other finger during this process. When the pressure chamber #2 is pressed, the pressure chamber #1 is pressed to prevent the solution entering from inlet #1. During this process, all of the solutions pass through the membrane filter, and an embedded absorbent pad under the membrane filter helps the solution pass through the holes on the membrane filter. By pressing and releasing the pressure chamber #1, the bacteria–MNP complex is filtered and remains on the membrane filter while unbound free MNPs are washed out, which generate a colorimetric signal on the filter membrane (Figure 2A,B). The bacteria–MNP complex, which has increased in size, may precipitate in inlet #1. To prevent this, the device was operated without delay right after dropping the solution into inlet #1. After the separation process is completed, the pressing and releasing operation at the pressure chamber #2 is repeated while the pressure chamber #1 is pressed. By this process, the gold enhancer solution from inlet #2 is introduced into the membrane filter based on the same principle as the previous procedure (Figure 2C,D). When the gold enhancer solution arrives at the membrane filter, the pressure chamber is no longer operated and incubated for signal enhancement. After the incubation, the gold enhancer solution is discharged by repeated pressing and releasing pressure chamber #2.

### 3.2. Effect of the Pore Size of the Membrane Filter on the Negative Control

**Figure 3:**
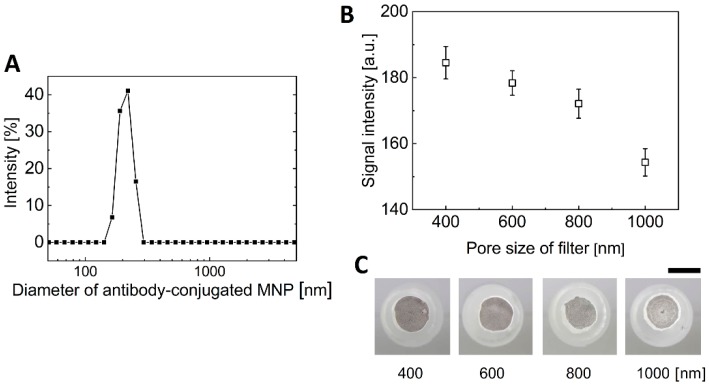
Effect of the pore size of the membrane filter on the negative control. (**A**) The size distribution of antibody-conjugated MNPs. (**B**) The negative control signal of four membrane filters with different pore sizes. (**C**) Images of the membrane filter. Scale bar = 3 mm.

Although immunomagnetic separation is a suitable technique for pretreatment to separate and concentrate target bacteria from the food matrix, it is impossible to detect bacteria because of coexisting unbound free MNPs. To use this immunomagnetic separation for the pathogen detection, unbound free MNPs should be separated from the bacteria–MNP complex. For the size-based separation between bacteria–MNP complex and free MNPs, MNPs with a diameter of 100 nm were used because the size of *E. coli* O157:H7 bacteria was from 500 nm to 2 μm. In addition, gold-coated MNPs were used for signal enhancement. As the aggregation of MNPs occurs inevitably in the conjugation process of the antibody, the size of antibody-conjugated MNPs was measured by dynamic light scattering (DLS) equipment. As shown in Figure 3A, the size of the antibody-conjugated MNPs was on average about 200 nm and did not exceed 300 nm. Therefore, membrane filters having a pore size bigger than 300 nm were used. Consequently, a polycarbonate membrane filter with a pore size of 400 nm, 600 nm, 800 nm, and 1 μm was embedded in a finger-powered device and the signal of the negative control was tested using only antibody-conjugated MNPs. As a result, the signal of a membrane filter with a large pore size was less than that of a membrane filter with a small pore size (Figure 3B,C). In addition, it was found that the overall negative control signal of all filters was large.

### 3.3. Comparison of E. coli O157:H7 Detection Signals According to the Pore Size Difference

**Figure 4:**
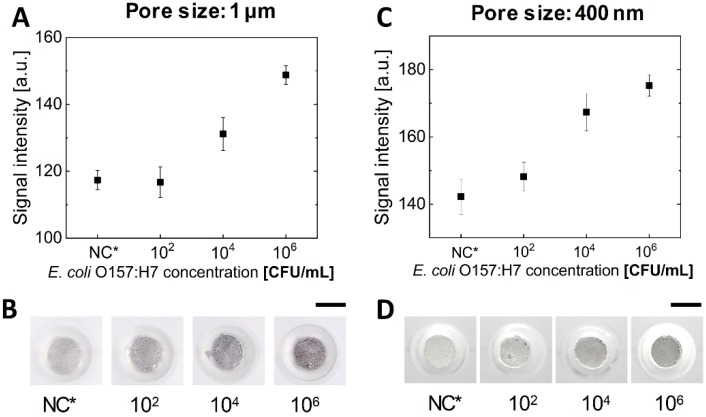
Colorimetric detection results of *E. coli* O157:H7 on a finger-powered microfluidic device when the filter membrane with different pore sizes was used. (**A**,**B**) 1 μm. (**C**,**D**) 400 nm. NC* indicates negative control. Scale bars = 3 mm.

As shown in the above section, the negative control signal of the membrane filter with a large pore size was smaller than that of the membrane filter with the smaller pore size. Although a membrane filter with a pore size of 1 μm showed a smaller negative control signal, it is necessary to consider the results for bacterial detection at an optimal condition. Therefore, in this study, membrane filters with different pore sizes from 400 nm to 1 μm were embedded in each finger-powered device and the signals according to the concentration of bacteria were analyzed. As a target analyte, *E. coli* O157:H7, a well-known foodborne pathogen, was used for this experiment. First, a membrane filter with a pore size of 1 μm was assessed (Figure 4A,B). At concentrations above 10^4^ CFU/mL, the signal was higher than the signal of the negative control. However, in the case of 10^2^ CFU/mL, the mean signal was not different from the negative control signal. In the case of membrane filters with a pore size of 400 nm, the signals were higher than the negative control signal at a concentration of 10^2^ CFU/mL or more (Figure 4C,D). Therefore, although the negative control signals were higher, the membrane filter with a pore size of 400 nm is better suited for detection than the membrane filter with a pore size of 1 μm because the concentration of 10^2^ CFU/mL was distinguishable. This result is due to the size of the bacteria. In the case of *E. coli* O157:H7, the width is generally 500 nm and the length is about 2 μm. Accordingly, in case of a pore size of 1 μm, some *E. coli* O157:H7 actually could pass through the membrane filter. However, in the case of a pore size of 400 nm, *E. coli* O157:H7 could not pass through the membrane filter.

### 3.4. Signal Enhancement Using Gold-Coated MNPs and Gold Enhancer

**Figure 5:**
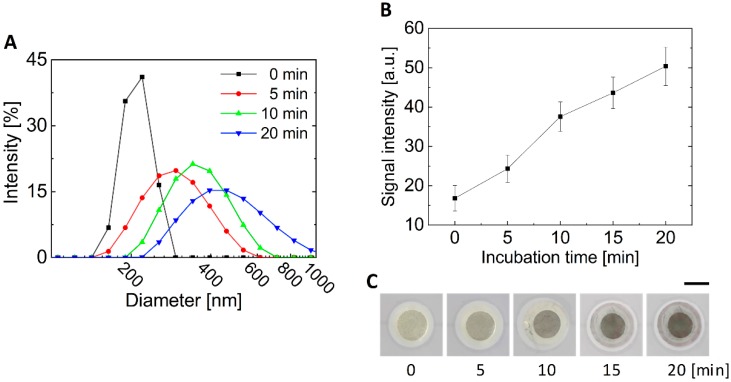
Effect of the gold enhancer on the colorimetric signal. (**A**) Size change of MNPs according to the incubation time in a gold enhancer solution. (**B**) Graph and (**C**) images showing the increasing colorimetric signal (negative control) in a finger-powered microfluidic device according to the incubation time with gold enhancer solution. Scale bar = 3 mm.

*E. coli* O157:H7 was detected by the colorimetric signal by the MNP-bound *E. coli* O157:H7 that could not pass through the membrane filter. In other words, it is not a direct signal from the bacteria, but an indirect signal by MNPs conjugated to the bacteria. Therefore, gold-coated MNPs can be used to enable amplification of the colorimetric signals. As the gold ions from the gold enhancer solution were attached to the surface of gold, the size of gold on the surface of MNPs was increased by the reaction with the gold enhancer, resulting in an amplification of the colorimetric signal. To characterize the increased size of gold-coated MNPs, the size of MNPs incubated with a gold enhancer solution for each time (5, 10, and 20 min) were measured using DLS equipment. As a result, the size of the gold-coated MNPs with a diameter of 200 nm was increased continuously to over 400 nm after 20 min (Figure 5A).

In addition, we observed the amplification of the colorimetric signals in the finger-powered device. For this experiment, gold-coated MNPs were injected into inlet #1, and were passed through the membrane filter by repeatedly pressing and releasing pressure chamber #1. At this time, pressure chamber #2 was pressed to close the valve to prevent gold-coated MNPs from going to inlet #2. For the signal enhancing test of the negative control signal, the gold enhancer solution was injected into inlet #2 and moved to the membrane filter by repeatedly pressing and releasing pressure chamber #2. When the gold enhancer solution reached the membrane filter, gold-coated MNPs remained on the filter (negative control signal) were incubated with the gold enhancer solution, stopping pressure chamber #2. The colorimetric signals were observed every 5 min for 20 min. As shown in Figure 5B,C, it was found that the signal continuously increased without saturation for 20 min, similar to the results of the size change of gold-coated MNPs using DLS. For the detection process (including conjugation process between bacteria and MNPs, pretreatment process, and signal enhancing process) within a total of 1 h, 10 min was set as the incubation time for the signal enhancement.

### 3.5. Detection of E. coli O157:H7 with Signal Enhancement

**Figure 6:**
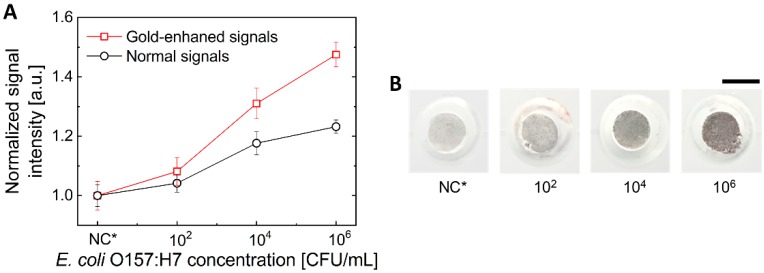
The results of colorimetric detection of *E. coli* O157:H7 on a finger-powered microfluidic device with signal enhancement. (**A**) The colorimetric signal was enhanced by incubation with a gold enhancer solution. NC* indicates negative control. (**B**) Images of an enhanced colorimetric signal on the membrane filter. Scale bar = 3 mm.

To demonstrate the detection of bacteria using the presented finger-powered microfluidic device, four different concentrations (10^2^, 10^4^, 10^6^, and 10^8^ CFU/mL) of *E. coli* O157:H7 were prepared. Antibody-conjugated gold-coated MNPs were added to 500 μL of an *E. coli* O157:H7 sample and incubated for 30 min for immunoreaction in the tube. Then, the MNPs were separated by placing a permanent magnet (NdFeB35) (Magtopia, Gumi, Korea) nearby the tube containing the solution for 15 min. And then, the MNPs were washed and retrieved with 100 μL PBS buffer. This prepared sample containing bacteria–MNP complex and unbound free MNPs was dropped in inlet #1. The sample was moved to the membrane filter and unbound free MNPs were washed out by repeatedly pressing and releasing pressure chamber #1. Thereafter, 100 μL of PBS buffer was dropped into inlet #1 and the membrane filter was washed with the PBS buffer by the operation of pressure chamber #1. After washing, the gold enhancer solution, dropped into inlet #2, was introduced to the membrane filter by operation of pressure chamber #2 with a finger. For signal enhancement, the sample was incubated in a gold enhancer solution for 10 min. After 10 min of incubation, the gold enhancer solution at the membrane filter was removed by the operation of pressure chamber #2. For the analysis, all signals were normalized to the average value of the negative control signal. As shown in Figure 6, the signal enhancement process resulted in a clear difference in the signal at a concentration above 10^2^ CFU/mL. However, at a concentration of 10^8^ CFU/mL, the sample solution could not be properly drained to the absorbent pad through the membrane filter. The pores of the membrane filter were plugged by too many bacteria. Therefore, the proposed device could detect *E. coli* O157:H7 in the concentration range from 10^2^ to 10^6^ CFU/mL.

## 4. Conclusions

In summary, *E. coli* O157:H7 was detected by the amplified colorimetric signal using a membrane filter-integrated finger-powered microfluidic device. After the immunomagnetic separation, the mixture of MNP-bound bacteria and unbound free MNPs were loaded on the membrane filter of the device by simply pressing and releasing the pressure chamber. The filtered MNPs bound to bacteria generate colorimetric signals on the membrane filter, and the gold enhancer solution was exploited for signal amplification as the gold-coated MNPs were used. After optimizing the pore size of the membrane filter and incubation time with gold enhancer solution, at least 10^2^ CFU/mL of *E. coli* O157:H7 was detectable within 1 h. Although the detection limit is not as low as in other studies based on fluorescence or SERS signal, it is meaningful noting that no other external signal readers or external pumping systems are required. It is further expected that the proposed device will play an important role in the on-site detection of foodborne pathogens by being integrated into an immunomagnetic separation apparatus.
